# Analysis of factors that affect the precision of the radiographic lateral femoral bowing angle using a three-dimensional computed tomography-based modelling technique

**DOI:** 10.1186/s13018-017-0588-x

**Published:** 2017-06-14

**Authors:** Ye-Ran Li, Yu-Hang Gao, Xin Qi, Jian-Guo Liu, Lu Ding, Chen Yang, Zheng Zhang, Shu-Qiang Li

**Affiliations:** Department of Orthopaedic Surgery, The First Hospital of Jilin University, Jilin University, Xinmin St 71, Chang Chun, China

**Keywords:** Lateral femoral bowing, Radiograph, Imaging, Computed tomography, Three-dimensional model, Total knee arthroplasty

## Abstract

**Background:**

Precise measurement of lateral femoral bowing is important to achieve postoperative lower limb alignment. We aimed to investigate factors that affect the precision of the radiographic lateral femoral bowing (RLFB) angle using three-dimensional (3D) models and whether the angle affects surgery design.

**Methods:**

Forty femurs in total were divided into two groups based on their preoperative RLFB angle. The flexion contracture angle, preoperative and postoperative RLFB angles, and intersection angle between the mechanical and anatomical axes were compared. The angle between the arc and sagittal planes, varus and valgus angles, and intersection angle between the mechanical and anatomical axes were measured on a 3D model.

**Results:**

There was no significant between-group difference in 3D model measurements of the angle between the arc and sagittal planes (*p* = 0.327). There was no significant difference between the mechanical and anatomical axes measured by both imaging modalities (*p* > 0.258). When the RLFB was >5°, the flexion contracture angle and radiographic femoral bowing angle were positively correlated (*r* = 0.535, *p* < 0.05). Distal femur varus and valgus angles significantly differed between the two groups (*p* = 0.01). After total knee arthroplasty, the radiographic femoral bowing angle decreased significantly. When the cases’ radiographic femoral bowing angle is larger and the angle between the arc and sagittal planes is smaller as measured in 3D models, the angle between the arc and coronal planes is larger.

**Conclusion:**

The radiographic femoral bowing angle does not reflect the actual size of lateral femoral bowing, does not greatly affect surgery design, and is greatly affected by flexion contracture deformity. A RLFB angle larger than 15° indicates real lateral femoral bowing.

## Background

Regaining lower limb alignment is crucial in total knee arthroplasty (TKA) [1, 2]. Preoperative planning based on long-leg weight-bearing anterior radiography aims to restore the alignment [3]. Precise femoral alignment relies on accurate femoral resection which depends on the angle between the mechanical and anatomical axes measured on a preoperative long-leg weight-bearing anterior radiograph. This angle can be affected by lateral femoral bowing; thus, miscalculation of lateral femoral bowing can directly lead to distal femoral resection error and subsequently femoral misalignment [4, 5].

Some researchers concluded that the lateral femoral bowing angle measured on radiographs can affect preoperative planning [5–8]. Akamatsu et al. reported that computed tomography (CT)-based measurements of lateral femoral bowing are much smaller than radiography-based measurements [7]. Some studies based on the latest three-dimensional (3D) technology indicated that surgery design should rely on both radiographic measurement of lateral femoral bowing and 3D measurements [6, 9]. However, few studies have investigated the factors affecting the difference between the lateral femoral bowing angle measured on radiographs and 3D models and whether femoral lateral bowing measured on radiographs affects the angle between the mechanical and anatomical axes measured on 3D models.

The present study aimed to compare the difference between radiographic lateral femoral bowing (RLFB) and 3D model-based lateral femoral bowing, to analyse the factors that affect the precision of the RLFB angle, and to determine whether the RLFB angle affects surgery design.

## Methods

### Study subjects

We prospectively enrolled patients who underwent primary knee arthroplasty in our department from June 2016 to September 2016. Patients who had a history of knee trauma or surgery and those with preoperative knee infection were excluded. The medical history and radiographic findings were recorded. The ethics committee of our institution approved this study, and all patients gave their informed consent to participate in this study. All of the patients enrolled in this study were assessed by physical examination (e.g. the degree of flexion contracture preoperatively). Patients were also evaluated using long-leg weight-bearing anterior radiographs preoperatively and postoperatively [8, 10], and long-leg CT (GE Discovery CT750 HD scanner, GE Healthcare, Waukesha, WI, USA) of the bilateral limbs with 5-mm thickness preoperatively. The mean and median RLFB angle was measured before and after the surgery. The patients were divided into two groups: those with a preoperative RLFB angle <5° were assigned to group A, and those with marked preoperative RLFB angle ≥5° were assigned to group B. The dividing method is also used in previous studies [3, 7].

### Imaging analysis

**Fig. 1:**
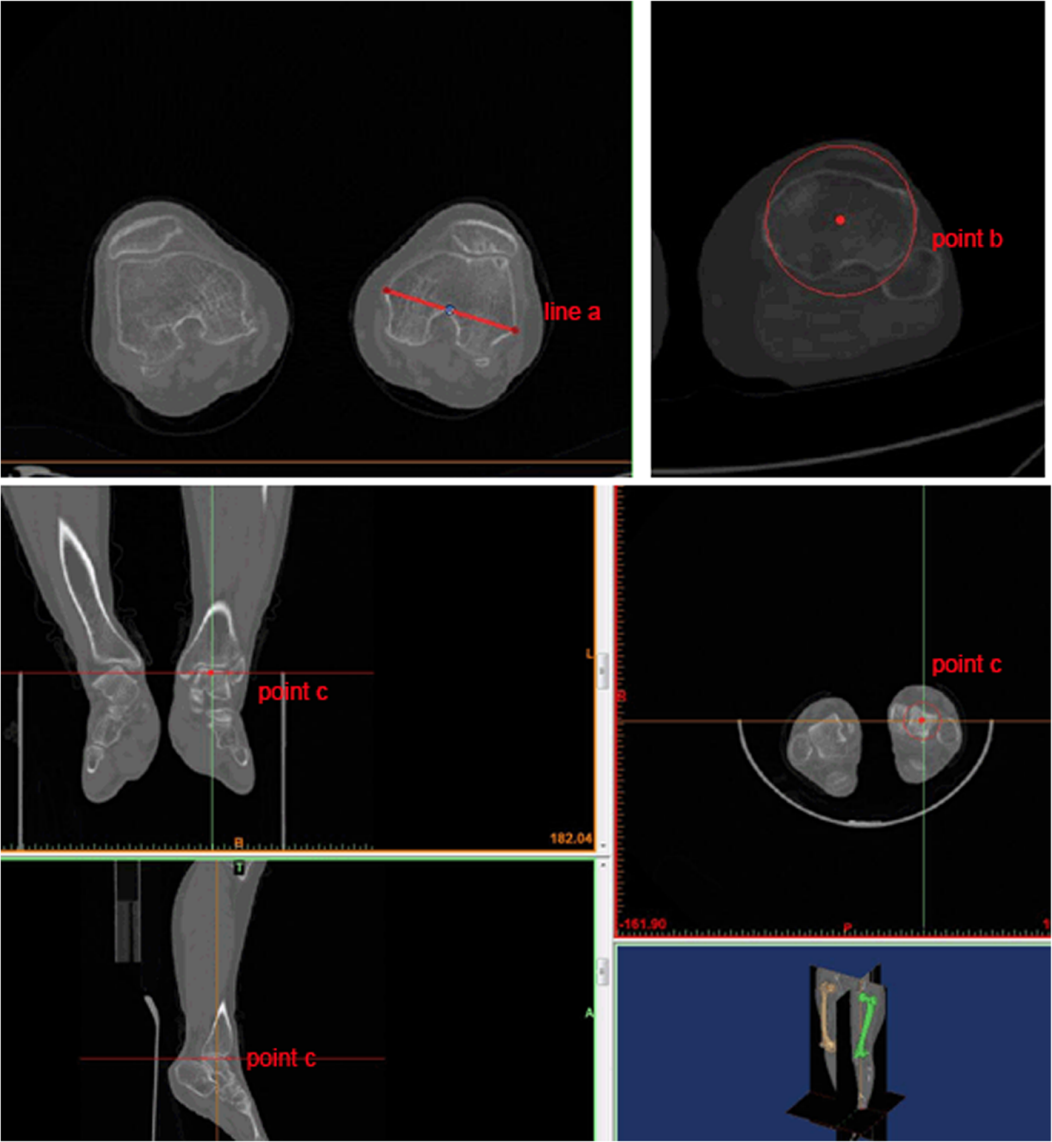
Line **a**: transepicondylar axis; Point **b**: the midpoint of the proximal tibia; Point **c**: distal tibia center point

**Fig. 2:**
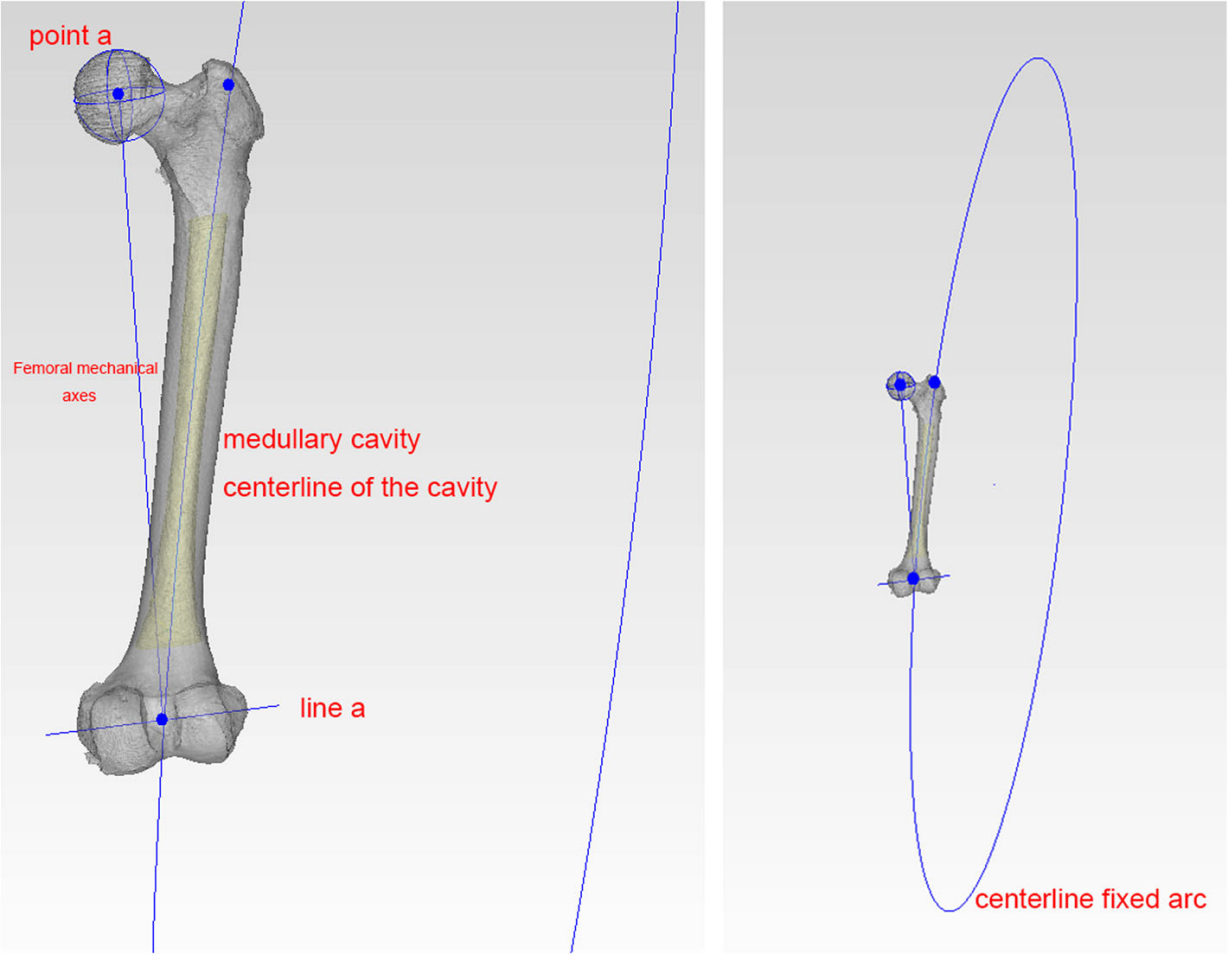
Point *a*: Femoral head point, the centre of a sphere fit to approximate the femoral head. Line *a*: transepicondylar axis. Femoral mechanical axis: The line across the midpoint of transepicondylar axis and femoral head point

**Fig. 3:**
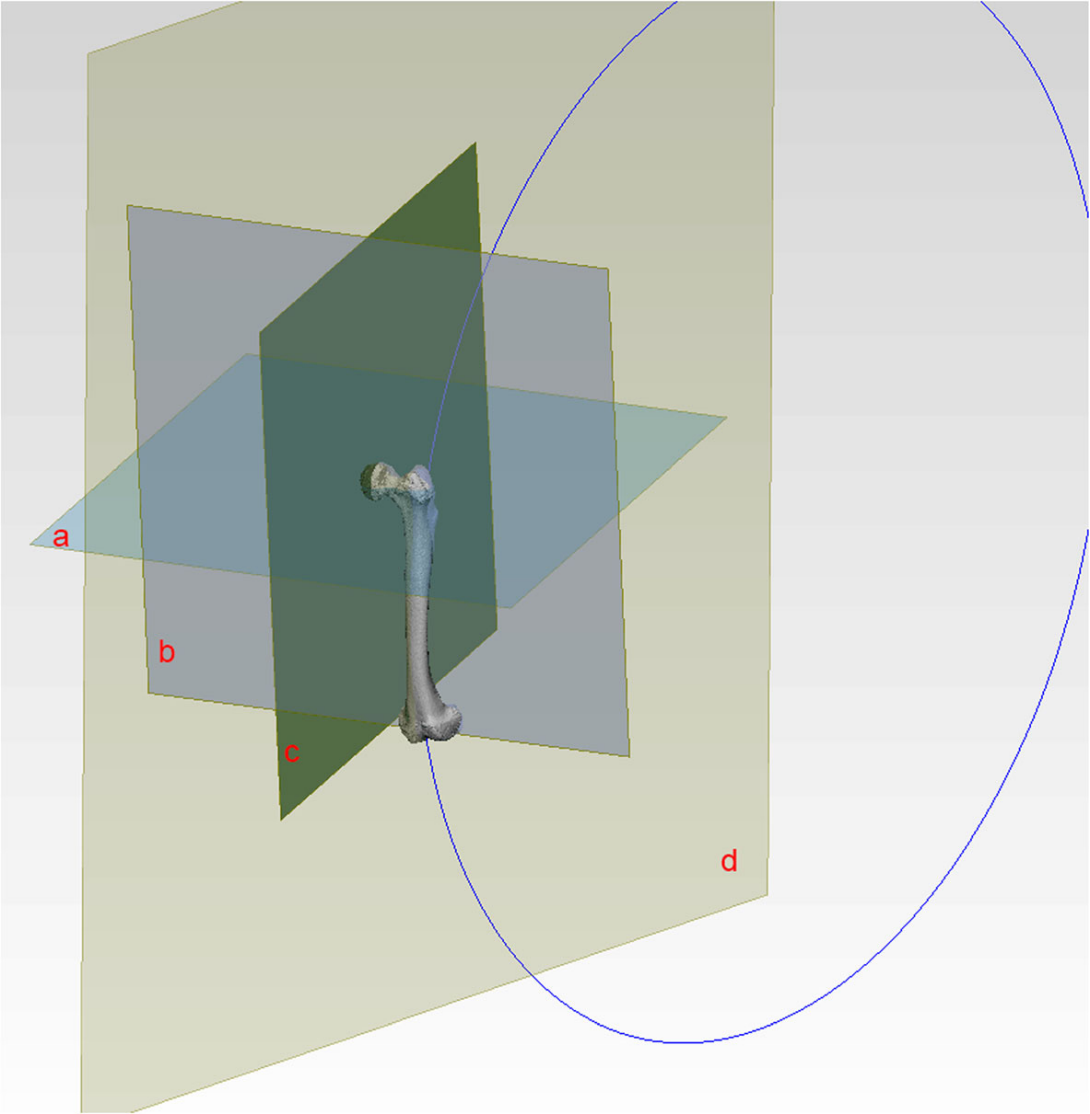
Plane *a*: axial plane; Plane *b*: coronal plane; Plane *c*: sagittal plane; Plane *d*: arc plane. Lower limb coronal plane: The plane formed by femoral transepicondylar axis and femoral head point. Lower limb sagittal and axial plane: perpendicular to coronal plane, respectively, and mutually

The RLFB angle [8, 10] and the angle between the mechanical axis and anatomical axis were measured on long-leg weight-bearing anterior radiographs preoperatively. All patients underwent full-length CT examination of the lower limbs before surgery, and the CT data were processed by Mimics 15 software (Materialise, Leuven, Belgium). The 3D data model of both lower limbs was obtained after modelling, and the model was anatomically measured by Mimics software. We defined a number of special anatomical landmarks by referring to previous literature [7, 11–15] (Figs. 1 and 2). With these landmarks in the 3D model, we could determine the coordinate system (Fig. 3). The measurement based on this coordinate system could effectively avoid the effect of flexion contracture, varus, valgus, or rotation. The following variables were measured in the 3D model coordinate system: the angle between the arc plane and sagittal plane and the angle between the mechanical axis and anatomical axis.

**Fig. 4:**
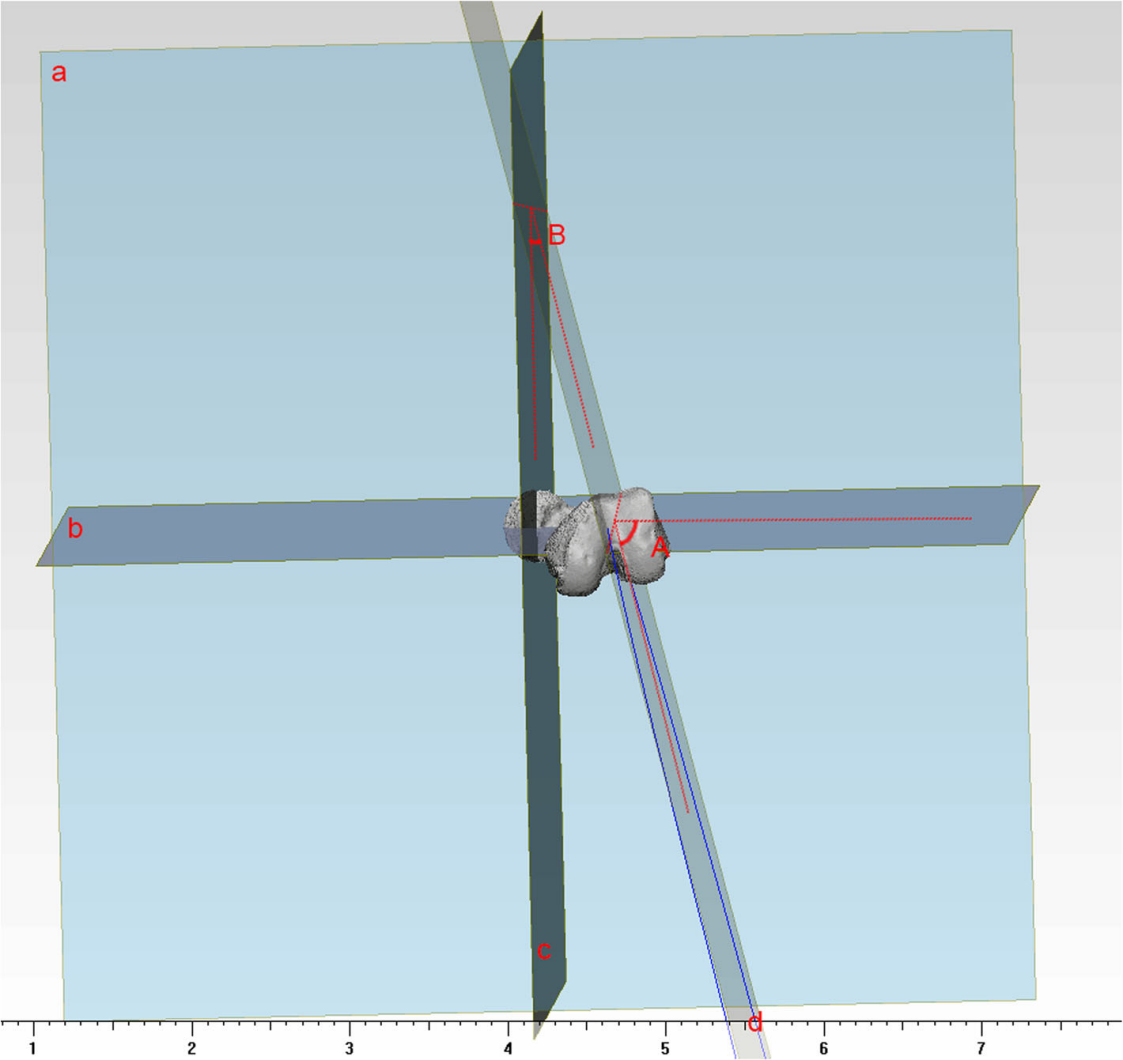
Plane *a*: axial plane; Plane *b*: coronal plane ; Plane *c*: sagittal plane ; Plane *d*: arc plane. Angle *A*: angle between arc plane and coronal plane; Angle *B*: angle between arc plane and sagittal plane. The arc plane is the formed by the fitted arc of central point and the femoral medullary cavity; thus, the arc is commonly influenced by both the femoral anterior bowing and lateral bowing. For the femur, the lateral bowing is often accompanied by anterior bowing; therefore, it is not appropriate to analyse each one of them at a time. The angle between the femoral sagittal plane and the arc plane (defined as 3D lateral femoral bowing index) can clearly reflect the lateral bowing degree of the femur. That is, the larger the femoral anatomy of the lateral bowing, the greater the 3D lateral femoral bowing index. While the angle between the arc plane and the coronal plane of the femur reflects the anterior arch degree of the femur. That is, the larger the anterior arch of the femur, the greater the angle between the arc plane and the coronal plane

Regarding the angle between the arc plane and sagittal plane (3D lateral femoral bowing index), the femoral medullary cavity centre line was established through the Mimics software, and the centre line was fitted into a complete arc. The plane was determined by the two intersections of the arc and the femur, and the centre of the arch was defined as the arc plane. The angle between the arc plane and sagittal plane was measured in the coordinate system (Fig. 4) [9].

Regarding the angle between the mechanical axis and the anatomical axis, the central point of the planes in CT of the one third isthmus of the medullary cavity was fitted into a straight line. This line was defined as the anatomical axis of the femur in the 3D model. The angle between the two lines projected onto the femoral coronal plane was defined as the angle between the mechanical axis and the anatomical axis in the 3D model.

Regarding the degree of varus and valgus deformity, the lower extremity model obtained from patients’ lower limb CT by using Mimics software was projected to the coronal plane, and then the “real” double lower extremity full-length “radiograph” was obtained without deviation caused by incorrect body position (e.g. internal and external rotation of the femur and knee flexion contracture). Then, the degree of varus and valgus deformity of the distal femur was measured by TRAUMA CAD 2.0 (Orthocrat, USA).

A high-volume senior orthopaedic surgeon performed all the surgeries in the present study. All the knee prostheses used in the present study were from the same company (PFC Sigma, Depuy, Warsaw, IN, USA).

The RLFB angle and radiographic angle between the mechanical axis and anatomical axis were also measured postoperatively. After the measurements were completed, the differences between the angles of the arc plane-sagittal plane and arc plane-coronal plane between the two groups; the difference in the degree of flexion contracture and the correlation between the RLFB angle and the degree of flexion contracture; the between-group difference in the angle of the distal end of the femur and its correlation with the RLFB angle; and the change in the RLFB angle before and after surgery were compared.

### Statistical analysis

*According to prior power analysis, this was the study size needed to meet the minimum requirement to achieve a power of 0.8 and an α value of 0.05*. The Shapiro-Wilk test was used to evaluate the normality of the data distributions. Data are expressed as median (range) and were compared using the Mann-Whitney *U* test. Correlations between two parameters were estimated by Pearson’s test when the data had a Gaussian distribution. Data with non-Gaussian distributions were compared between groups using the Kruskal-Wallis test. A *p* value <0.05 was considered statistically significant. All statistical analyses were performed using SPSS Statistics 22 (IBM Corporation, Armonk, NY, USA).

## Results

A total of 30 patients were enrolled in this study. Three of them were male and the remaining 27 were female. The mean age was 65.7 ± 7.16 (range, 51–83) years. Forty femurs were evaluated (left, 19; right, 21); 6 were from male patients and 34 were from female patients. The median of the RLFB angle was 4.95°. According to the preoperative RLFB angle, 20 femurs were included in the group with an RLFB angle <5° and 20 were included in the group with an RLFB angle >5°. There were no significant differences in the baseline characteristics between the two groups (Table 1). When comparing the angle of the sagittal plane and arc plane, no significant between-group difference was found (*p* = 0.327) (Table 1).

**Table 1:** Baseline characteristics between group A and group B

	Group A(<5°)	Group B(>5°)	*p*
Gender (m/fm)	4/21	2/13	1.000
Age (years)	64.95 ± 7.83	66.55 ± 6.52	0.231
Height (cm)	162.55 ± 6.67	159.60 ± 7.84	0.231
Weight (kg)	66.70 ± 8.70	65.02 ± 7.93	0.883
BMI (kg/cm^2^)	25.33 ± 3.91	25.21 ± 2.95	0.889

A significant difference between groups was considered for *p* < 0.05

The radiographic and 3D model-based angle between the mechanical axis and anatomical axis was 6.44° ± 1.54° and 6.84° ± 1.33°, respectively. The absolute value of the difference between the two angles was not significantly correlated with the plain RLFB angle (*p* = 0.258). The difference between the angle of the two sets of mechanical axes and anatomical axes was not significantly different between the two groups (*p* = 0.314).

The relationship between the two groups according to the RLFB angle and the flexion contracture angle was compared. When the RLFB angle was >5°, the flexion contracture angle had a strong linear positive correlation with the RLFB angle (*r* = 0.535, *p* < 0.05), that is, the bigger the RLFB angle, the larger the flexion contracture angle. When the RLFB angle was <5°, there was no strong correlation between the flexion contracture angle and the RLFB angle. The mean ± SD and median values of the flexion contracture angle of the group with an RLFB angle >5° were greater than those of the group with an RLFB angle <5° (11.20 ± 6.97 and 8.92 vs 9.59 ± 7.99 and 5.39, respectively).

When the RLFB angle was >5°, there was no linear correlation between the RLFB angle and varus and valgus deformity(*r* = 0.281). However, a significant difference in varus and valgus deformity between the two groups was found (*p* = 0.03) (Table 2).

**Table 2:** Relationship of groups A and B according to arc plane and sagittal plane angle, flexion contracture and varus and valgus deformity

	Angle between arc plane and sagittal plane	Flexion contracture	Varus and valgus deformity
<5° group A	19.05 ± 7.85	9.59 ± 7.99	2.20 ± 1.83
>5° group B	24.39 ± 14.07	11.20 ± 6.97*	4.17 ± 2.20**
*p*	0.327	0.221	0.03

*A significant difference between groups was considered for *p* < 0.05. When the radiographic RLFB angle is greater than 5°, the flexion contracture angle has strong linear positive correlation with the RLFB angle (*r* = 0.535, *p* < 0.05)

Comparing the angle of sagittal plane and arc plane, it is found that there is no statistical difference between the two groups (*p* = 0.327)

**When RLFB is greater than 5°, it has no linear correlation with the varus and valgus deformity, but there are statistical differences in the varus and valgus deformity between two groups (*p* = 0.03)

For patients whose RLFB angle was >5°, the mean ± SD and median angle between the arc and sagittal plane were 24.39 ± 14.07 and 20.40, respectively. This group of cases was divided into two groups using the arc-sagittal plane angle of 20° as an interface. When the RLFB angle was larger and the angle between the arc and sagittal plane smaller, the angle between the arc and coronal plane is larger (77.01 ± 5.91 vs 52.38 ± 11.95), and the two sets of data were statistically significant (*r* = −0.954, *p* = 0.01).

**Fig. 5:**
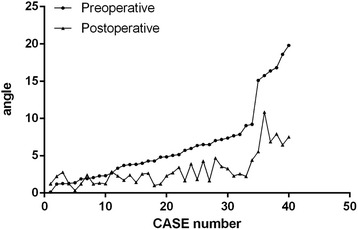
Preoperative radiographic lateral femoral bowing angle is 5.52 ± 4.56, 4.57, while the postoperative radiographic lateral femoral bowing angle’s average is 3.01 ± 1.99, 2.60. When flexion contracture and varus and valgus deformity are corrected through surgery, the average postoperative RLFB angle becomes significantly lower than the preoperative RLFB angle. The RLFB decreases after surgery, which further confirms that the larger RLFB angle is caused by flexion contracture and varus and valgus deformity

By measuring the preoperative and postoperative RLFB angle, the flexion contracture deformity and the varus and valgus deformity were corrected by surgery. The mean ± SD and median postoperative RLFB angle were 3.01 ± 1.99 and 2.60, respectively, which were significantly lower than the mean ± SD and median preoperative RLFB angle (5.52 ± 4.56 and 4.57, respectively). The patients’ RLFB angle disappeared after surgery (Fig. 5).

In the group with an RLFB angle >5°, a notable larger femoral bow angle >15° was found in six cases. In these six cases, the femoral bow angle remained greater than 5° after surgery.

## Discussion

A total of 40 femurs from 30 patients were evaluated in the present study and were divided into two groups in accordance with the preoperative RLFB angle, where an RLFB angle of 5° was used as the cut-off value to divide both groups, *in accordance with previous publications* [3, 7]. A preoperative RLFB angle >5° could result in a wrong judgment regarding force alignment; therefore, special attention should be paid to the bone section intraoperatively [3, 7, 16].

According to the results of our research, the RLFB angle cannot reflect the real size of the lateral femoral bowing. Compared with previous research, the 3D method measurement for the difference between the mechanical axis and anatomical axis is more accurate. In the present study, there was no between-group difference in the angle between the mechanical axis and anatomical axis measured by 3D CT and radiography, indicating that the RLFB angle does not have a large effect on the surgery design.

The flexion contracture is a major factor that affects the RLFB angle. Patients with a larger preoperative flexion contracture deformity have a larger difference in the femoral bow angle. When the RLFB angle was >5°, a positive linear correlation was found between the flexion contracture angle and the RLFB angle, that is, the larger the flexion contracture angle, the larger the femoral bow angle.

The varus and valgus deformity at the distal femur can also affect the RLFB angle, but its effect is not greater than that of flexion contracture. There was no significant correlation between the varus and valgus angle and the RLFB angle, but the varus and valgus angle is significantly different between the two groups (*p* = 0.03).Therefore, the effect of the varus and valgus angle of the distal femur on the RLFB angle should still be acknowledged. In the present study, the varus and valgus angle of the distal femur was effectively measured on the femoral coronal plane. This measurement method avoids inaccurate measurements on a plain radiograph caused by incorrect position and flexion contracture.

Concerns about the effect of the RLFB angle on surgical design have been previously reported. Abdelaal et al. showed that after CT reconstruction, the larger lateral femoral bowing angle measured by CT had an effect on the surgery design [16], but was not correlated to the radiographic examination. Their study showed that the femoral bowing is expressed through the axial plane measurement and in multiple sections. Compared with our research methods, their methods cannot show the lateral femoral bowing’s overall situation very well. Shi et al. measured the femoral bowing based on preoperative radiographs and concluded that the RLFB angle can effectively guide surgery [17]. Although the measurement of femoral bowing on radiographs is a common practice, it cannot eliminate the effects caused by flexion and varus and valgus deformity.

Lee et al. suggested that the femoral bow affects the angle measurement of the mechanical and anatomical axes by misleading the positioning of the anatomical axis of the femur, which greatly affects the surgery design [5]. Their measurement, however, is based on radiographs and, in the preoperative period, often leads to shooting position inaccuracy because of the flexion contracture deformity and varus and valgus deformity of the distal femur. Our research is based on a 3D model, and the measurement of the angle between the mechanical axis and the anatomical axis rules out the influential factors on the quality of radiographs. No significant difference in the 3D measurement of the “real” angle between the mechanical axis and anatomical axis was found between large and small RLFB angle groups.

The present study also found that in the cases in which the RLFB angle was bigger and the 3D measurement of the angle between the sagittal and arc plane was smaller, the angle between the coronal and arc plane was larger and the difference was statistically significant. We believe that flexion contracture or deformity causes lateral femoral rotation, which can lead to greater projection of coronal femoral bowing in radiography, and seemingly, the RLFB appears to be larger. When both the RLFB angle and 3D measurement of the angle between the sagittal and arc plane are larger, the angle between the coronal and arc plane becomes smaller.

When flexion contracture and varus and valgus deformity are corrected through surgery, the average postoperative RLFB angle becomes significantly lower than the preoperative RLFB angle. The RLFB disappears after surgery, which further confirms that the larger RLFB angle is caused by flexion contracture and varus and valgus deformity.

In six cases, the RLFB angle was >15° and the RLFB angle remained greater than 5° after surgery indicating that an RLFB angle greater 15° preoperatively prompts the existence of a real lateral femoral bowing angle, and the postoperative residual bowing observed on radiography will be ≥5°. Therefore, we believe that when the RLFB angle is >15°, the patient has real lateral femoral bowing.

The unique methods applied in this study are mainly embodied in the following three aspects. First, through 3D printing design technology, a coordinate system was established, in which a circle could be drawn on the basis of the centre line of the femoral marrow cavity. The size of the sagittal angle-arc plane was used to present femoral lateral bowing, which allows for quantitative analysis of lateral femoral bowing and reflects the extent of the bow lateral femoral stereoscopic and related measurement. Second, through the comparison of the angle between the mechanical and anatomical axes with 3D measurement and traditional radiograph measurement, we judged whether the larger RLFB affects the preoperative surgery design. As a result of the high precision of measurement with the 3D model, which can fully reflect the real angle between the mechanical axis and anatomical axis, the degree of surgical correction can be planned more accurately. Finally, through the comparison of the preoperative and postoperative RLFB angle, the flexion contracture and varus and valgus deformity were corrected and the RLFB angle disappeared postoperatively, indicating that these two factors affect the RLFB angle which further verifies our findings.

The present study has some limitations. First, the sample size was small; however, the radiographic lateral bowing angle data were widely distributed; thus, enough cases with large radiographic lateral bowing were included. Second, this was a single-centre study. The conclusions of the present study should be confirmed in a multi-centre study with a larger sample size. Third, we only explored the main influencing factors such as flexion contracture deformity and varus and valgus deformity of the distal femur; other factors might be discovered through further study.

## Conclusion

In conclusion, radiographic measurement cannot accurately reflect the size of the lateral femoral bowing angle. The RLFB angle is greatly affected by flexion contracture deformity as well as varus and valgus deformity of the distal femur. Although the RLFB angle is larger than that measured by 3D CT, it does not affect the angle between the mechanical and anatomical axes and the surgery design. A large RLFB angle and small sagittal and arc angle prompt femoral rotation, which can lead to greater projection of radiographic coronal femoral bowing and, seemingly, a larger lateral bowing angle. A large 3D lateral femoral bowing index and radiographic lateral femoral bowing angle indicate a real large lateral femur bowing angle. An RLFB angle >15° indicates real lateral femoral bowing.

## Abbreviations

3D: Three-dimensional
CT: Computed tomography
RLFB: Radiographic lateral femoral bowing
TKA: Total knee arthroplasty
