# Erratum to Analysis of factors that affect the precision of the radiographic lateral femoral bowing angle using a three-dimensional computed tomography-based modelling technique

**DOI:** 10.1186/s13018-017-0603-2

**Published:** 2017-07-17

**Authors:** Ye-Ran Li, Yu-Hang Gao, Xin Qi, Jian-Guo Liu, Lu Ding, Chen Yang, Zheng Zhang, Shu-Qiang Li

**Affiliations:** Department of Orthopaedic Surgery, The First Hospital of Jilin University, Jilin University, Xinmin St 71, Chang Chun, China

## Erratum

In the original publication of this article [1] were one value error in the abstract and two sentences in the body of the article were in italics instead of in normal font.

Error 1:

Distal femur varus and valgus angles significantly differed between the two groups (*p* = 0.01).

Should be read instead:

Distal femur varus and valgus angles significantly differed between the two groups (*p* = 0.03).

Error 2:

*Statistical analysis According to prior power analysis, this was the study size needed to meet the minimum requirement to achieve a power of 0.8 and an α value of 0.05.*

Should be read instead:

Statistical analysis According to prior power analysis, this was the study size needed to meet the minimum requirement to achieve a power of 0.8 and an α value of 0.05.

Error 3:

A total of 40 femurs from 30 patients were evaluated in the present study and were divided into two groups in accordance with the preoperative RLFB angle, where an RLFB angle of 5° was used as the cut-off value to divide both groups, *in accordance with previous publications* [3, 7].

Should be read instead:

A total of 40 femurs from 30 patients were evaluated in the present study and were divided into two groups in accordance with the preoperative RLFB angle, where an RLFB angle of 5° was used as the cut-off value to divide both groups, in accordance with previous publications [3, 7].

The original article was updated to rectify these errors.
